# Feasibility of functional magnetic resonance imaging of ocular dominance and orientation preference in primary visual cortex

**DOI:** 10.1371/journal.pcbi.1007418

**Published:** 2019-11-04

**Authors:** Marilia Menezes de Oliveira, James C. Pang, Peter A. Robinson, Xiaochen Liu, Mark M. Schira

**Affiliations:** 1 School of Physics, University of Sydney, New South Wales, Australia; 2 Center for Integrative Brain Function, University of Sydney, New South Wales, Australia; 3 QIMR Berghofer Medical Research Institute, Herston, Queensland, Australia; 4 School of Psychology, University of Wollongong, Wollongong, New South Wales, Australia; Oxford University, UNITED KINGDOM

## Abstract

A recent hemodynamic model is extended and applied to simulate and explore the feasibility of detecting ocular dominance (OD) and orientation preference (OP) columns in primary visual cortex by means of functional magnetic resonance imaging (fMRI). The stimulation entails a short oriented bar stimulus being presented to one eye and mapped to cortical neurons with corresponding OD and OP selectivity. Activated neurons project via patchy connectivity to excite other neurons with similar OP in nearby visual fields located preferentially along the direction of stimulus orientation. The resulting blood oxygen level dependent (BOLD) response is estimated numerically via the model’s spatiotemporal hemodynamic response function. The results are then used to explore the feasibility of detecting spatial OD-OP modulation, either directly measuring BOLD or by using Wiener deconvolution to filter the image and estimate the underlying neural activity. The effect of noise is also considered and it is estimated that direct detection can be robust for fMRI resolution of around 0.5 mm, whereas detection with Wiener deconvolution is possible at a broader range from 0.125 mm to 1 mm resolution. The detection of OD-OP features is strongly dependent on hemodynamic parameters, such as low velocity and high damping reduce response spreads and result in less blurring. The short-bar stimulus that gives the most detectable response is found to occur when neural projections are at 45 relative to the edge of local OD boundaries, which provides a constraint on the OD-OP architecture even when it is not fully resolved.

## Author summary

Ocular dominance (OD) and orientation preference (OP) cells of the visual cortex are numerically simulated to investigate the feasibility of their *in vivo* determination via functional magnetic resonance imaging (fMRI) in humans. A short oriented bar with OD-OP features is mapped to the primary visual cortex and the blood oxygen level dependent (BOLD) response is numerically estimated via a spatiotemporal hemodynamic response function model. The model predicts the BOLD response from a given neural activity and vice versa. Our results show that direct OD-OP detection from BOLD is feasible for a resolution of 0.5 mm, while detection via Wiener deconvolution can be attained to resolutions from Δ*x* ≈ 0.125 − 1.0 mm.

## Introduction

The overall field of view of the eyes is mapped in a topographic fashion to the primary visual cortex (V1) via a one-to-one retinotopic mapping [1–4]. V1 cells are characterized by two response properties, ocular dominance (OD) and orientation preference (OP) [5–9]. OD columns are associated to binocularity, i.e., the convergence input of left and right eyes onto single cells, that underlie depth perception [1, 10]. On the other hand, OP is related to cells that respond best to a specific stimulus orientation, which changes linearly in patches of size 0.5 to 1 mm [10–12]. The purpose of the present work is to determine whether functional magnetic resonance imaging (fMRI) can be used to detect and map these feature preferences in V1 and to determine the required spatial resolution to do so.

**Fig 1:**
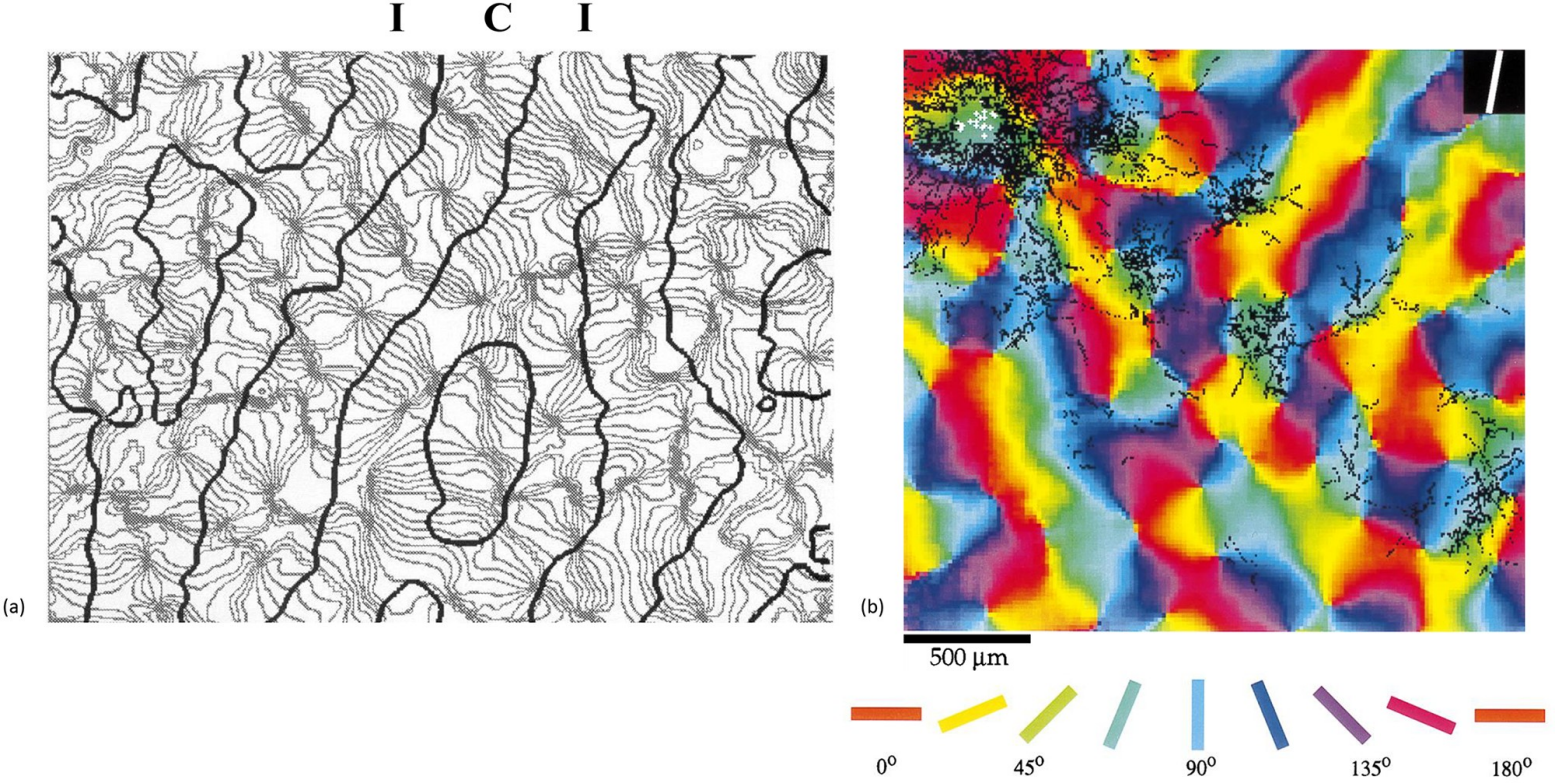
Ocular dominance (OD) and orientation preference (OP) in V1. (a) Combined OD and OP data from macaque. The black contours separate ipsilateral (I) and contralateral (C) OD, while the gray lines are contours of constant OP [12]. (b) Patchy connectivity in tree shrew striate cortex. The white dots represent sites of anterograde biocytin staining injection and the black dots show the bouton distribution (excited areas) to which these points project, showing that projection is chiefly to points of similar OP (indicated by color, as labeled below the figure) beyond the immediate VF projection. The white line in the black square on the top right corner represents the OP direction of the stimulation [13].

A joint representation of OD and OP from a macaque is shown in Fig 1(a). This demonstrates that all OD-OP features are mapped to within about 1 mm of any given point. Most OPs rotate continuously through ±180° as the azimuth changes relative to a nearby singularity to form a “pinwheel”; pinwheel centers tend to lie near the center of OD stripes [4, 11–13]. A region that contains all feature preferences is termed a hypercolumn, and the part of the field of view mapped to a given hypercolumn is termed a visual field (VF). V1 contains patchy horizontal connections that preferentially connect neurons with similar OP in neighboring VFs, as seen in Fig 1(b); moreover, these projections extend furthest along an axis corresponding to the OP direction, with the overall zone of projections being elongated along the OP direction [13].

Invasive experiments, such as using optical imaging techniques based on voltage-sensitive dyes in nonhuman species [10, 12, 14–16], have been the only way to analyze OD and OP. The same technique has been performed to postmortem human brain but only with monocular vision [15]. In the latter, they confirmed the existence of OD columns in all analysed individuals, with morphology and spacing variability due to genetic factors. Hence, due to limitations of invasive techniques, it is important to find noninvasive ways to image OD-OP maps in normal human subjects.

fMRI is the dominant noninvasive technique to analyze organization patterns in the human cerebral cortex. However, limitations to current fMRI spatial resolution makes it difficult to map cortical structures smaller than 1 mm in size [15]. Improvements in the resolution of fMRI to around 0.8 mm in 7 tesla (7 T) scanners places fMRI on the threshold of being able to image combined OD-OP features [17–19]. By modeling 7 T fMRI, Chaimow et al. [20] concluded that imaging the OD-OP architecture is optimal at a resolution of around 0.8 mm due to increasing noise at finer resolutions. They considered pattern, voxel size, fMRI point-spread, and noise characteristics, but did not undertake any deconvolution of the hemodynamic response because of the lack of a suitable physiologically based model of its mechanisms. In particular, direct imaging cannot extract the neural origins of the blood oxygen level dependent (BOLD) response, nor can neural inhibition and excitation be distinguished from function-specific fMRI signals [21].

Most clinical MRI scanners have field strengths of 1.5 T or 3 T, while major neuroimaging centers mostly use 3 T or 7 T [22], with a few as high as 20 T [23]. The spatial resolution of 3 T scanners is approximately (1 mm)^3^ for anatomical images and approximately (2 mm)^3^ for functional scans based on BOLD or perfusion contrast signals. On the other hand, 7 T devices increase anatomical resolution to (0.5 mm)^3^ and fMRI resolution to about (1 mm)^3^ [22]. Ultra-high fMRI resolution is possible if pushing the limits and using optimization methods, in that way isotropic resolution of 0.5 mm can be reached. fMRI measures the BOLD signal during a hemodynamic response to neural activity [21, 24], which includes propagating waves that spread the BOLD signal [25] and thus reducing the accuracy with which it can be used to directly track the underlying neural activity.

Recent physiology-based modeling has enabled the link between neural activity and BOLD to be made quantitative, including spatial spreading and temporal dynamics [25, 26]. Conversely, this model has been used as the basis of Wiener deconvolution to estimate the underlying neural signal from the BOLD response, thereby enabling sharper images to be obtained even in the presence of noise [27, 28].

This work aims to explore the feasibility of detecting OD and OP in V1 using fMRI, either directly or via deconvolution, and to determine the short-bar stimulus orientation that maximizes detectability within a particular OD column organization, laying the basis for testing experimental designs of optimal stimuli. To achieve this, the above mentioned quantitative hemodynamic model is employed [24–28] to calculate the response to a patchy neural activity [29]. The model is used to estimate the BOLD response to the spatially patchy neural activity that results from visual stimuli, then the imaging requirements for OD-OP detection are estimated. Finally, deconvolution is applied to sharpen the images and the resulting detection criteria are determined [25–28].

The paper is organized as follows. Section Theory and methods explore the feasibility of stimulating and imaging OD and OP columns. The section discusses patchy neural activity in V1, the spatiotemporal hemodynamic response function (stHRF) that enables prediction of the BOLD response to arbitrary neural activity, deconvolution of the underlying driving neural activity from arbitrary BOLD data, and simulated data used for testing the methods. Section Results discusses the results of the work, showing the simulated BOLD response to the neural activity and the most easily way to be detected for potential detection of OD and OP columns using fMRI. Finally, Sec. Summary and discussion summarizes the results of the work and highlights the implications of the study.

## Methods

This section describes our proposed neural activity and its retinotopic mapping to V1. Then the model of BOLD dynamics is summarized. The stHRF of the model is demonstrated, which is used to predict the BOLD response to arbitrary neural activity and to deconvolve the neural activity from arbitrary BOLD data. Finally, the patchy neural response is described. The codes needed to implement the methods in this section can be found at https://github.com/BrainDynamicsUSYD/Project_ODOP.

### Patchy propagator

We consider a visual stimulus presented to one eye in the field of view that consists of a short oriented bar. The stimulus excites neurons with OP similar to the bar orientation. This neural activity is then propagated to other cortical neurons of similar OP features via patchy connections of the type seen in Fig 1(b), this patchy neural activity then drives the BOLD response. This relation can be approximated as the product of an overall oriented envelope function and a local periodic modulation. We write the envelope as an oriented elliptic Gaussian centered at a source point **r′** = (*x*′, *y*′) and projecting to other points **r** = (*x*, *y*) for a preferred OP angle *φ*(*x*, *y*), with [29]

$$\begin{array}{cc} G(r,r^{\prime }) & =\exp[-\frac{1}{2}(\frac{x_{g}^{2}}{\delta_{x}^{2}}+\frac{y_{g}^{2}}{\delta_{y}^{2}})], \end{array}$$ (1)

$$\begin{array}{c} x_{g}=\left(x-x^{\prime }\right)\cos\left[\varphi \left(x,y\right)\right]+\left(y-y^{\prime }\right)\sin\left[\varphi \left(x,y\right)\right], \end{array}$$ (2)

$$\begin{array}{c} y_{g}=-\left(x-x^{\prime }\right)\sin\left[\varphi \left(x,y\right)\right]+\left(y-y^{\prime }\right)\cos\left[\varphi \left(x,y\right)\right], \end{array}$$ (3)

where *δ*_*x*_ and *δ*_*y*_ are the spatial widths in *x*_*g*_ and *y*_*g*_, respectively. The modulation of this envelope due to the patchy connectivity is approximated as having a spatial period of *a* in the orthogonal *x*- and *y*-directions, which is the width of a unit cell. Here, the unit cell represents a small portion of V1 with width of 2 mm and that includes relevant OD-OP features. The product of this modulation and the Gaussian function in Eq (1) yields

$$\begin{array}{cc} G(r,r^{\prime }) & =\exp[-\frac{1}{2}(\frac{x_{g}^{2}}{\delta_{x}^{2}}+\frac{y_{g}^{2}}{\delta_{y}^{2}})]\\ & \times \{\frac{1}{2}\cos[k_{x}(x-x^{\prime })]+1\}\{\frac{1}{2}\cos[k_{y}(y-y^{\prime })]+1\}, \end{array}$$ (4)

where *k*_*x*_ = *k*_*y*_ = 2*π*/*a* are the spatial frequencies in the *x*- and *y*-directions.

**Fig 2:**
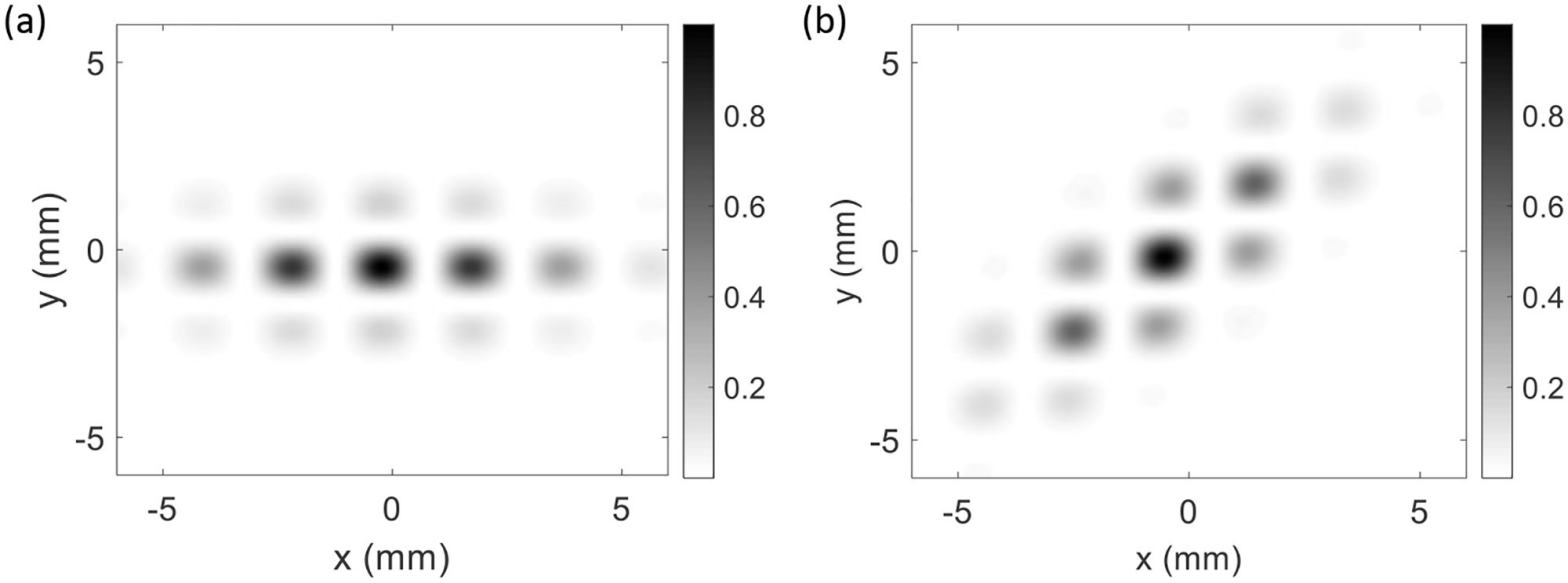
Patchy propagator model from Eq (4) with unit cell with *a* = 2 mm, spatial widths of *δ*_x_ = 2.83 mm and *δ*_y_ = 1 mm, and two OPs. (a) *φ* = 0°, (b) *φ* = 45°. The color bar shows the strength of connection from highest (black) to lowest (white).

Fig 2 shows examples of the patchy propagator in Eq (4) for two values of OP for stimuli that map to the same central unit cell. We see a maximum intensity at the central point and patchy projections that fall off over a few millimeters within an envelope with a 3:1 aspect ratio, and the long axis is in the direction of the OP, consistent with Fig 1(b).

### Mapping to V1

To make this first analysis tractable, we approximate the OD columns in Fig 1(a) by strips with fixed width and straight boundaries. The visual cortex can be approximated as a square hypercolumn within each hypercolumn of 1 mm dimension for the left and right eyes [29]. In this approximation, a unit cell is chosen to include both left and right OD, with two pinwheels of opposite handedness in each—this unit cell can be repeated to tile the entire region of V1. Each pinwheel has a range from 0° to 180° [30]. At first, one unit cell is built starting from the point (0, 0) at the center; the top-right pinwheel is then centered at $\left(x_{0}/a,y_{0}/a\right)=\left(\frac{1}{2},\frac{1}{2}\right)$. The OP angle *φ*(*x*, *y*) in this quadrant ranges from 0 to *π*, (*x*/*a*, *y*/*a*) range from −1 to 1 in *x* and *y*, with

$$\begin{array}{c} \varphi \left(x,y\right)=\frac{1}{2}[\tan^{-1}(\frac{y-y_{0}}{x-x_{0}})+\pi ]. \end{array}$$ (5)

**Fig 3:**
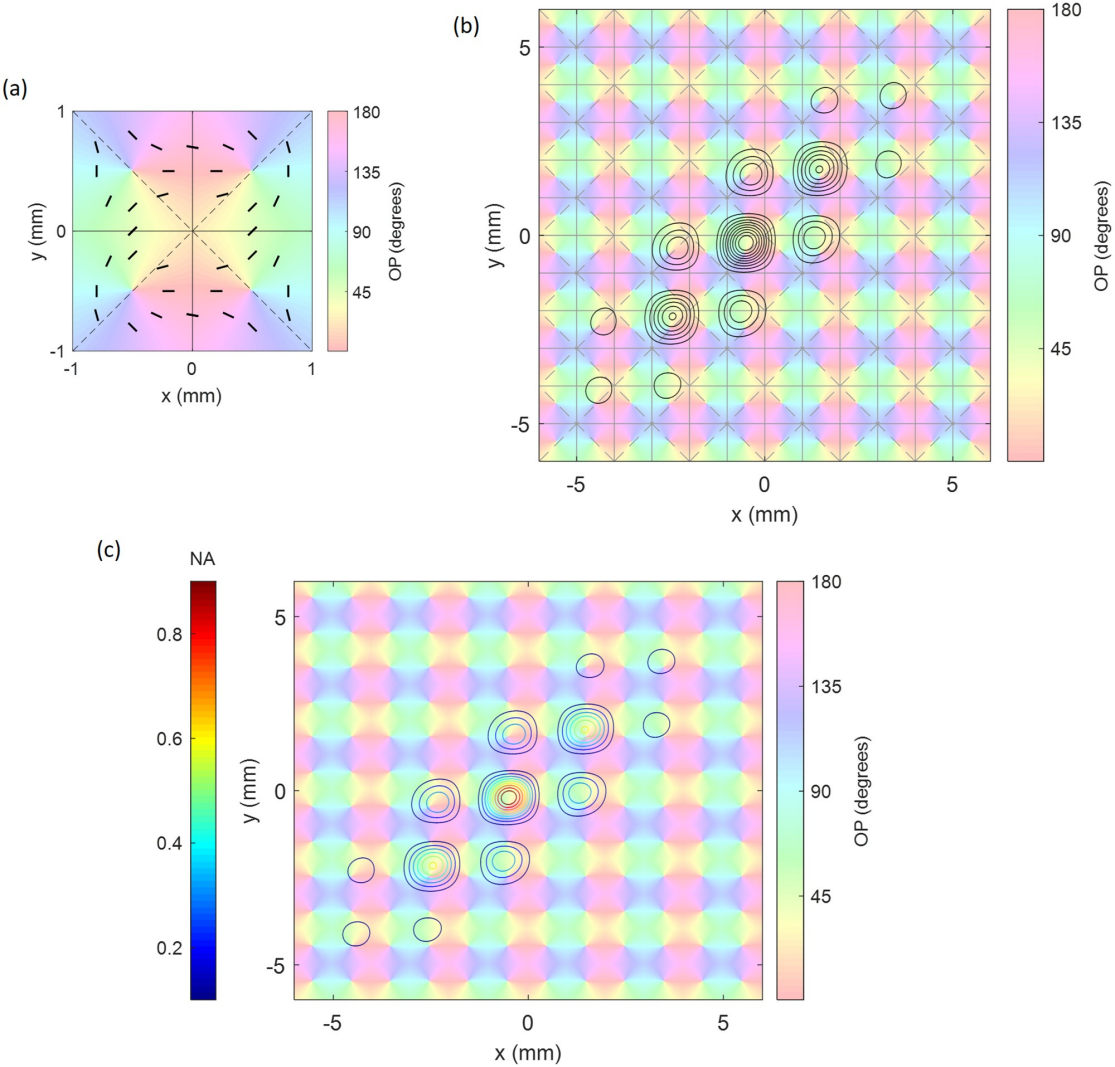
OD-OP feature preference map as a function of position (x,y) in V1. (a) Unit cells with left-eye (L) and right-eye (R) OD stripes are labeled, with OP angles from 0° to 180° superimposed, as indicated by the colors. The black bars highlight the OP at various points. (b) Set of 36 unit cells, with the black contours showing patchy connections from the central unit cell for OP angle of 45°. (c) Same set of 36 unit cells, and the patchy propagator of neural activity (NA) shows the magnitude and intensity of neural response as colored contour lines (left color bar).

The other pinwheels are obtained by reflecting the first one across the *x*- and/or *y*-axes. Fig 3(a) shows the resulting OD-OP map for a single unit cell, with orientations highlighted by short bars. Fig 3(b) shows the patchy projections of activity from a horizontal stimulus in the central unit cell, centered in the region near the boundary between the left-eye (L) and right-eye (R) OD columns. Note that the peaks of the pattern are shifted slightly from the peaks of the cosine function in Eq (4) by the overall shape of the Gaussian envelope.

The patchy projections yield responses that embody local neural tuning curves. As a stimulus bar is rotated, the peak response and its projections, as seen in Fig 3(c), shift across V1 to the corresponding OP locations. Thus, the excitation of neurons at any given point rises smoothly as the bar approaches their OP, then falls off again as it deviates; note that the width of the peaks is adjusted to match experimental tuning curve widths of around 40° full width at half maximum [31].

### BOLD response

Here, we describe the stHRF used to produce the BOLD response to a stimulus-induced neural activity. Afterwards, we describe the Wiener deconvolution method that approximately recovers the neural activity from the BOLD response even in the presence of noise.

#### BOLD response estimation

The hemodynamic model that produces the stHRF makes three main approximations to physically model spatiotemporal hemodynamics: (i) cortical tissue is considered as a porous elastic medium; (ii) neural activity and hemodynamics are treated on a two-dimensional (2D) sheet; and (iii) dynamics are quantified as local averages.

**Fig 4:**
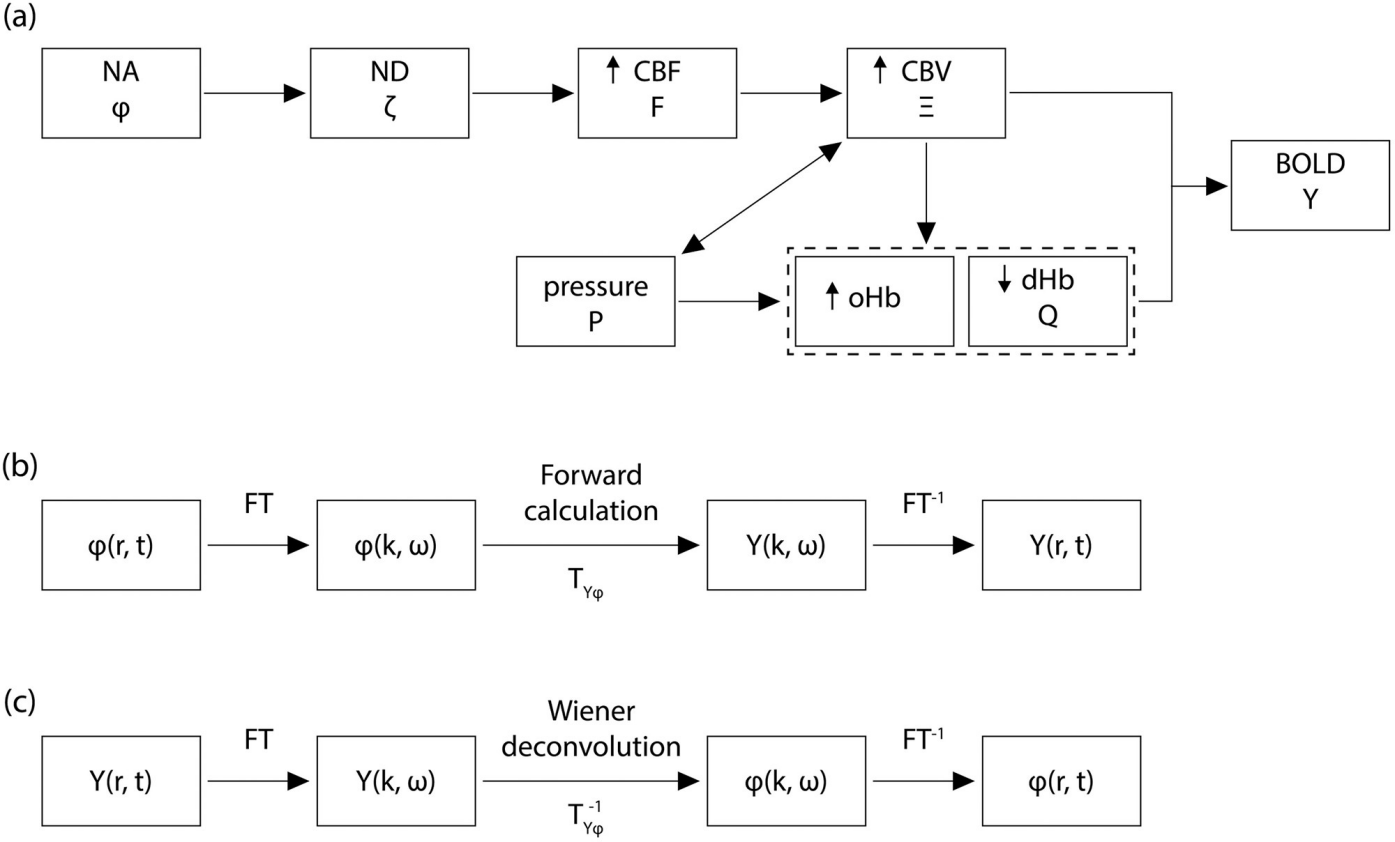
Flowcharts describing the stHRF physical processes in the model and the deconvolution method. (a) Estimation of the BOLD response from a neural activity (NA), including the intermediary physiological processes. The enclosed dashed box in oHb and dHb denotes that they are almost simultaneously affected by the other processes. ND denotes neuroglial drive. (b) Summary of the mathematical formulation for the BOLD estimation from NA. (c) Summary of the mathematical formulation to recover the NA from BOLD. For panels b and c, *T* is the transfer function and *T*^−1^ is its inverse, while FT denotes the Fourier transform and FT^−1^ denotes its inverse.

Using the above approximations, the model quantifies spatiotemporal changes of the physiological processes involved in the generation of the BOLD response in terms of coupled differential equations. The physical processes in the model are illustrated in the flowcharts in Fig 4(a) and 4(b) and can be summarized as follows. An increase in neural activity *ϕ*(**r**, *t*) at position **r** and time *t* on the cortical tissue activates surrounding astrocytes via neurotransmitters. The activated astrocytes produce a response called the neuroglial drive *ϵ*(**r**, *t*) that affects nearby vasculature, leading to an increase in cerebral blood flow (CBF) *F*(**r**, *t*). This increases cerebral blood volume (CBV) *Ξ*(**r**, *t*), which leads to deformation of the surrounding tissue, exerting pressure *P*(**r**, *t*) on nearby vessels. Increases in blood flow and volume lead to increases in the concentration of oxygenated hemoglobin (oHb), consequently decreasing the local concentration of deoxygenated hemoglobin (dHb) *Q*(**r**, *t*). Finally, these overall changes in CBV and dHb concentration contribute to changes in the BOLD signal *Y*(**r**, *t*). Solving the equations of the model allows the calculation of the stHRF that relates the BOLD signal and the input neural activity. Full details and derivations of equations of the model are described in the relevant literature [25, 26, 28, 32].

Experiments have shown that the hemodynamic response is approximately linear for low-amplitude neural responses [25–27, 32–35]. Hence, the BOLD signal *Y*(**r**, *t*) can be expressed as the convolution of the stHRF *H*(**r**, *t*) and neural activity *ϕ*(**r**, *t*) such that

$$\begin{array}{c} Y(r,t)=H(r,t)⊗\phi (r,t), \end{array}$$ (6)

where ⊗ is the spatiotemporal convolution operator. Taking the Fourier transform of Eq (6) then yields

$$\begin{array}{c} Y(k,\omega )=H(k,\omega )\phi (k,\omega ), \end{array}$$ (7)

where **k** is the spatial frequency, *ω* is the temporal frequency, and *Y*(**k**, *ω*), *H*(**k**, *ω*), and *ϕ*(**k**, *ω*) are the Fourier transforms of *Y*(**r**, *t*), *H*(**r**, *t*), and *ϕ*(**r**, *t*), respectively. The Fourier transform can be applied here because the response is short range and the system boundaries are sufficiently far away that their effects can be neglected.

The stHRF in frequency space *H*(**k**, *ω*) is the transfer function that can be directly derived from the model (*H*(**k**, *ω*) = *T*_*Y*,*ϕ*_(**k**, *ω*)) [28]. It describes the change in BOLD signal per unit change in neural activity at the same **k** and *ω*. Hence, if the neural activity is known, the response can be calculated by taking the inverse Fourier transform of Eq (7)

$$\begin{array}{c} Y\left(r,t\right)={FT}^{-1}[H(k,\omega )\phi (k,\omega )], \end{array}$$ (8)

where *FT*^−1^ is the inverse Fourier transform.

#### Neural activity estimation

The above model can predict the BOLD signal if *ϕ*(**r**, *t*) is accurately known. However, fMRI experiments only give direct information about the BOLD signal and not the neural activity. The inverse problem of estimating neural activity *ϕ*(**r**, *t*) from BOLD *Y*(**r**, *t*) can be addressed by implementing a deconvolution method using the model’s transfer function *H*(**k**, *ω*) [27, 28].

Deconvolution can be implemented via the following steps, as summarized in Fig 4(c):

(i) Fourier transform the BOLD signal *Y*(**r**, *t*) to obtain *Y*(**k**, *ω*);
(ii) use the stHRF in frequency space *H*(**k**, *ω*) to construct a Wiener filter (discussed below) that can robustly obtain *ϕ*(**k**, *ω*); and
(iii) take the inverse Fourier transform of *ϕ*(**k**, *ω*) to get the neural activity *ϕ*(**r**, *t*) in coordinate space.

The advantage of this deconvolution method is that it can be applied to an arbitrary BOLD signal to recover the neural activity. In principle, at step (ii), we could get the neural activity *ϕ*(**k**, *ω*) directly from BOLD *Y*(**k**, *ω*) by reversing Eq (7) and using the inverse of the transfer function *H*(**k**, *ω*) instead of a Wiener filter. However, for real data with intrinsic noise, this method would lead to corrupted solutions because noise effects at high spatial and temporal frequencies where noise exceeds the signal would be disproportionately amplified [27, 28, 36, 37]. One way to solve this inverse problem is to use a Wiener filter whose design is informed by the likely noise structure of the raw signal [38, 39]. This filter is used in many deconvolution methods and is mathematically described as

$$\begin{array}{c} D\left(k,\omega \right)=\frac{1}{H(k,\omega )}\frac{{\left|H\left(k,\omega \right)\right|}^{2}}{{\left|H\left(k,\omega \right)\right|}^{2}+{\left|NSR\left(k,\omega \right)\right|}^{2}}, \end{array}$$ (9)

where *NSR* is the noise-to-signal ratio. Assuming that the noise is white and the signal is an impulse function in space and time, *NSR*(**k**, *ω*) can be approximated by a constant *σ* [27, 28, 39, 40], whose size we estimate in the next subsection. The effect of the term |*NSR*(**k**, *ω*)|^2^ in Eq (9) is to suppress the effects of noise at large **k** and *ω*.

### Simulated data

The main objective of the work is to explore the feasibility of detecting OD and OP features in fMRI experiments. To do this, we first simulate the neural response to bar stimuli taking into account OD and OP, as described by Eq (4) and in Fig 2. Then, the model is used to predict the resulting BOLD response at a given spatial resolution Δ*x*; this becomes our simulated BOLD data. White noise of rms (root mean square) amplitude *σ* is added to the BOLD signal to account for experimental noise, which depends on the spatial resolution Δ*x*. The simulated data are then deconvolved to estimate the neural activity and test the method’s accuracy as a function of OD, OP, and spatial resolution.

**Fig 5:**
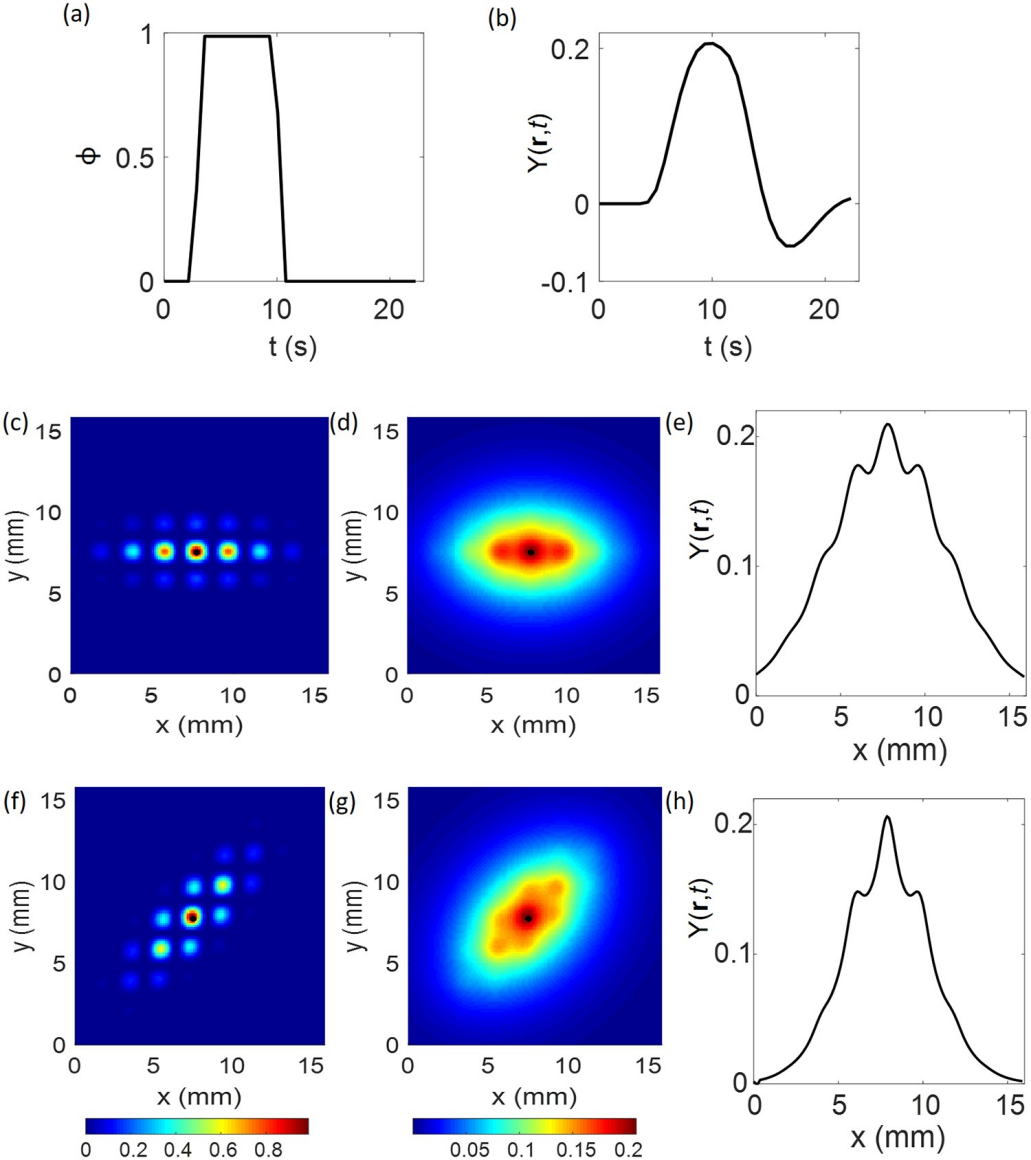
Neural activity and BOLD response. (a) Profile of neural activity *ϕ*. (b) BOLD response *Y*(**r**, *t*) vs. time, at the most strongly stimulated location (position at the black point near the center of panels c, d, f, and g). (c) Neural activity for OP of 0°. (d) BOLD response for OP of 0°. (e) Profile of BOLD response for OP of 0° at *t* = 9.7 s. (f) Neural activity for OP of 45°. (g) BOLD response for OP of 45°. (h) Profile of BOLD response for OP of 45° at *t* = 9.7 s. *x* and *y* are the dimensions in mm. For panels (c) to (h), the resolution used is 0.125 mm. The color bar shows the magnitude of neural activity and BOLD response.

The simulations considered here have one short bar stimulus mapped to V1 via retinotopic mapping and patchy connections pattern of activity as in Fig 2 and maximum duration of 23 s. In the first 3 s, there is no stimulation; from 3 s to 10 s, a step function stimulus of unit magnitude is ‘on’; after which it is ‘off’, as shown in Fig 5(a).

Employment of a spatial resolution Δ*x* corresponds to filtering the signal plus noise in Fourier space. We adopt the following combined procedure to incorporate the correct level of noise and filter the image to the corresponding voxel resolution, following [41] and [42]:

(i) Construct a random Gaussian spatial white noise field at very fine spatial resolution—at least as fine as the finer resolution considered in this work.
(ii) Fourier transform this field into the wavenumber (*k*) space.
(iii) Filter using a rectangular window that imposes the restriction |*k*| < *k*_*c*_ = 2*π*/2Δ*x*, where *k*_*c*_ is the cutoff point. This smooths the signal at a resolution of Δ*x* without imposing particular voxel locations, related to the grid of voxels. It corresponds to a sinc-function smoothing in coordinate space.
(iv) Inverse Fourier transform into the coordinate space.
(v) Normalize according to the work of [41] and [42] so that the signal-to-noise ratio for task-based measurements is 200(Δ*x*/1mm)^2^ for voxels of size (Δ*x*) × (Δ*x*) × 3 mm, as discussed in those papers. This value is used as our estimate of *σ* in the Wiener filter.
(vi) Add the noise to each simulated spatial BOLD signal and repeat steps (ii)–(v) for the combined signal to obtain the filtered BOLD signal at resolution Δ*x* with corresponding noise in coordinate space.
(vii) Discretize to voxels of size (Δ*x*) × (Δ*x*) in the image plane by choosing a specific grid of voxel locations and assigning BOLD values to each from the smoothed but unpixelated result from (vi).

## Results

In this section, numerical fMRI simulations are performed to explore the feasibility of detecting OD and OP structures. Another key question is whether OD and OP can be detected directly from the BOLD response, or whether deconvolution methods need to be applied, especially in the presence of measurement noise. Also, we seek to determine what short-bar stimulus will produce the most easily detectable response, and how its OP is related to the local OD architecture.

### BOLD response prediction

The stimulus at V1 considered here is the one described in Sec. Simulated data and Fig 5(a). It is normalized for values between 0 and 1 for simplicity of calculation. The resulting BOLD response *Y*(**r**, *t*) in Fig 5(b) shows that, upon presentation of the stimulus, a large peak response is observed followed by a post-stimulus undershoot before returning to baseline. This stereotypical shape is consistent with the temporal profile of point-wise temporal hemodynamic response functions widely used in neuroimaging studies [43].

Fig 5(c) shows the neural activity corresponding to cells with OP of 0° plotted with a resolution of 0.125 mm and no noise. The position of strongest stimulation is marked by the black dot. From this position, the stimulus is propagated to nearby VFs in the OP direction, in this case 0°, exciting other cells with OP of 0°. Thus, the resulting neural activity has a structure that looks like a string of pearls. The stHRF is then convolved with this neural activity and the simulated BOLD response is generated. The BOLD response for this OP of 0° is shown in Fig 5(d). With the parameters used in this case, some modulation is only visible near the center of the BOLD response. However, in general, the response is smooth and it is not possible to differentiate each separate region with OP of 0° position, unlike in the neural activity. The spatial profile of the BOLD response is also analyzed. The profile considered is from a line passing through the position of highest magnitude of the BOLD response and in the relevant OP direction. The profile at *t* = 9.7 s is shown in Fig 5(e). The BOLD modulation is *ξ* ≈ 6%, as defined by

$$\begin{array}{c} \xi =\frac{\frac{1}{2}\left(P_{+}+P_{-}\right)-M}{\frac{1}{2}\left(P_{+}+P_{-}\right)+M}, \end{array}$$ (10)

where *P*_+_ is the central maximum of the BOLD response, *P*_−_ is the adjacent maximum along the main axis of the response, and *M* is the intervening minimum; if there is no such minimum *ξ* = 0. An analogous definition of neural modulation *ϵ* yields a value of ≈ 97%.

The neural activity for an OP of 45° is shown in Fig 5(f) at resolution of 0.125 mm without noise. For that case, the projection of neural activity is in the 45° direction. Similarly, the stHRF technique is applied and the BOLD response is estimated, as shown in Fig 5(g). Evident modulation is visible near the origin of the neural activity, but in general, the response has no visible modulation. The profile for this BOLD response is shown in Fig 5(h) and has *ξ* ≈ 10%, whereas *ϵ* = 100%. For an OP of 90°, the modulation is similar to that for OP of 0°; i.e., *ξ* ≈ 6%. Similarly, the modulation for OP of 135° is equal to that for an OP of 45°; i.e., *ξ* ≈ 10%.

When the orientations of patchy projections are diagonal relative to OD column boundaries (i.e., at OPs of 45° and 135° in the present case) the distance of the center of the neural activity to its neighbors is multiplied by a factor of $\sqrt{2}$ because of the angle of the stimulus projection pattern relative to the OD stripes. This strengthens the modulations relative to cases with 0° and 90°. Note that for other OD architectures the stripes may not be vertical and the OP with optimal detectability will be the one whose patchy projections in V1 are at 45° to the local OD boundaries.

### Dependence on fMRI resolution

**Fig 6:**
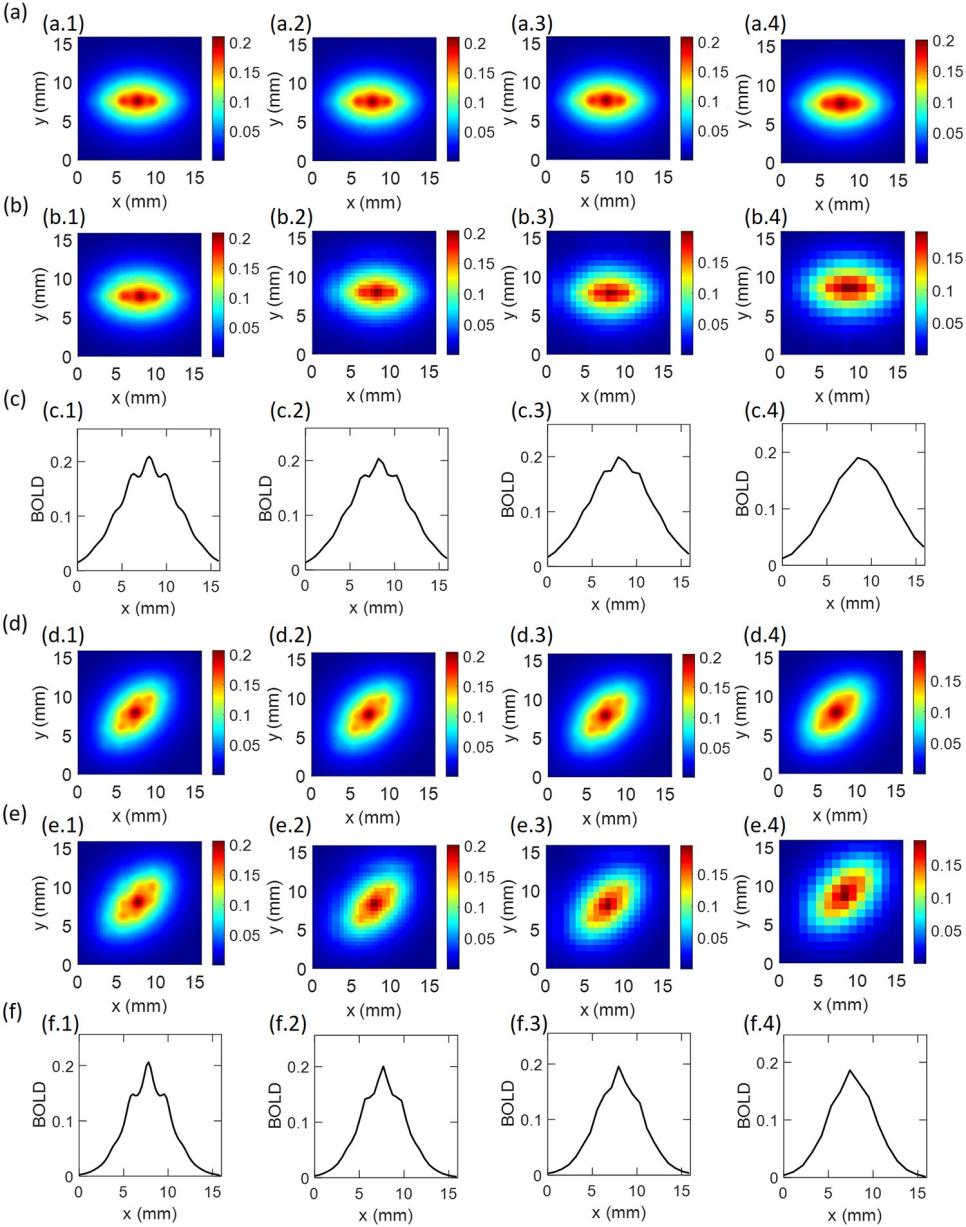
Coarse-grained BOLD responses for stimulus orientations of (a-c) 0° and (d-f) 45°. (a,d) Smoothed BOLD response. (b,e) Pixelated BOLD response. (c,f) BOLD response profile versus distance along the OP direction. The columns are arranged from left to right to show results for resolutions of 0.25 mm, 0.50 mm, 0.75 mm, and 1.0 mm, respectively. For all panels, the profiles are taken at *t* = 9.7 s.

The results up to now have been computed at a spatial resolution of 0.125 mm. In Fig 6, we examine the dependence on actual fMRI resolution for Δ*x* = 0.25 − 1.0 mm. Fig 6(a)–6(c) show the results for OP of 0°, with Fig 6(a) showing the smoothed, unpixelated results in the absence of noise. Fig 6(b) shows the same results for a specific choice of pixel locations, while Fig 6(c) shows BOLD profiles along the main axis of the plots in Fig 6(b). Fig 6(d)–6(f) show the corresponding results for OP of 45°. We see that the spatial OD-OP modulation remains visible in the BOLD images at 0.25 mm resolution with *ξ* ≈ 6% for OP of 0° and *ξ* ≈ 10% for OP of 45°. At 0.50 mm resolution, only the OP of 0° shows modulation, with *ξ* ≈ 5%; however, resolutions coarser than that show no modulation and no resolved OP regions.

### Deconvolution to estimate neural activity

**Fig 7:**
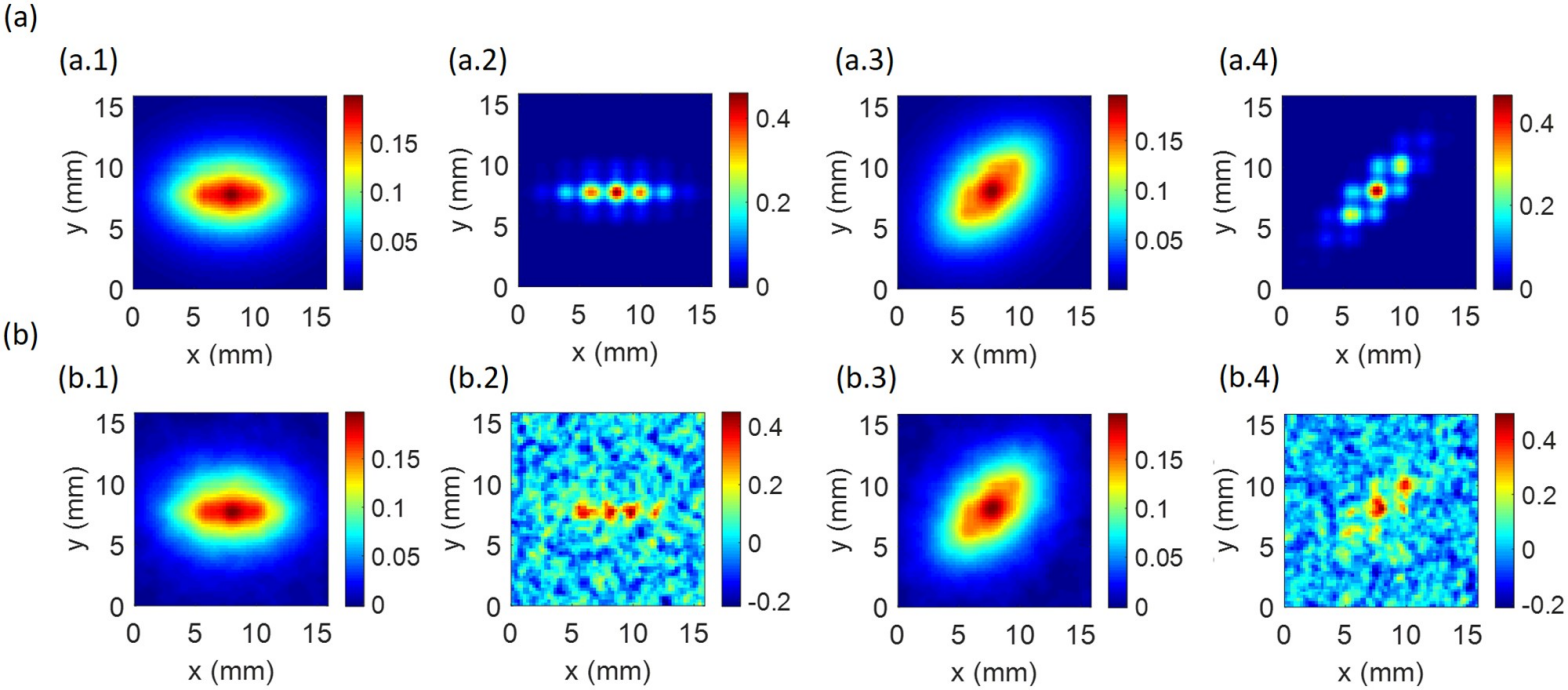
Deconvolution to recover neural activity. (a) Deconvolution method without noise. (a.1) BOLD-Wiener with *σ* of 0.06 for OP of 0°. (a.2) Deconvolved neural activity from panel a.1. (a.3) BOLD-Wiener with *σ* of 0.06 for OP of 45°. (a.4) Deconvolved neural activity from panel a.3. (b) Deconvolution to recover neural activity with white noise. White noise varies with voxel volume (as described in Sec. Simulated data). (b.1) Noisy BOLD-Wiener for OP of 0°. (b.2) Deconvolved neural activity from panel b.1. (b.3) Noisy BOLD-Wiener for OP of 45°. (b.4) Deconvolved neural activity from panel b.3. For all panels, the resolution used is 0.25 mm.

This section describes the results of the deconvolution of the BOLD response to estimate the underlying neural activity, as shown in Fig 7 for resolution of 0.25 mm. Fig 7(a.1) shows an example of the Wiener deconvolution from Sec. Neural activity estimation [Eq (9)] using *σ* = 0.06 for OP of 0°. Here, the Wiener filter is applied to the BOLD response from Fig 5(d) to reduce extraneous noise. Because there is no measurement noise in the original image, we use *σ* = 0.06 in this case. For brevity, we term this type of filtered BOLD signal ‘BOLD-Wiener’. From there, the Wiener deconvolution method described in Sec. Neural activity estimation is applied and the neural activity is recovered, as shown in Fig 7(a.2). For this case, where no noise is included in the BOLD signal, the neural activity is recovered accurately and it is possible to see the patches of excited cells centered on the OP direction of 0° in this example, with neural activity modulation *ϵ* ≈ 98%. Modulation is also visible for coarser resolutions, when Δ*x* is from 0.5 to 1.0 mm, *ϵ* varies from 64% to 37%. The BOLD-Wiener signal is shown for an OP of 45° in Fig 7(a.3). Once again, the Wiener deconvolution is employed and the neural activity is recovered, as shown in Fig 7(a.4). As expected, the strongly stimulated cells are along a line on the OP direction of 45° and it is possible to differentiate each activity peak from its neighbors in adjacent VFs without the blurring seen in the BOLD signal due to hemodynamic spreading. For these ideal cases (without noise), the reconstructed neural activity shows *ϵ* ≈ 99%. For Δ*x* from 0.5 to 1.0 mm, *ϵ* varies from 88% to 40%.

The second row of Fig 7 is the same as the first, except that noise is included in BOLD, as described in Sec. Simulated data, and the same level of noise is used in the Wiener filter. The signal-to-noise ratio thus varies in proportion to (Δ*x*)^2^ and has a value of 200 for the present task-based conditions at Δ*x* = 1 mm. Fig 7(b.1) and 7(b.3) show the BOLD response with noise and then Wiener filtered (we call this signal ‘noisy BOLD-Wiener’) for OPs of 0° and 45°, respectively, with 0.25 mm resolution; while Fig 7(b.2) and 7(b.4) show the neural activity estimated by deconvolving the noisy BOLD-Wiener signal. Despite the noise, the Wiener filter successfully localizes the neural activity much better than the BOLD signal and yields inferred neural activity modulations of *ϵ* ≈ 46% and *ϵ* ≈ 80% for OPs of 0° and 45°, respectively, compared to no modulation in the BOLD signal itself. That means, even if the BOLD image shows no resolved OD-OP features, deconvolution is able to resolve modulation of the undetected neural activity.

### Sensitivity to physiological parameters

**Fig 8:**
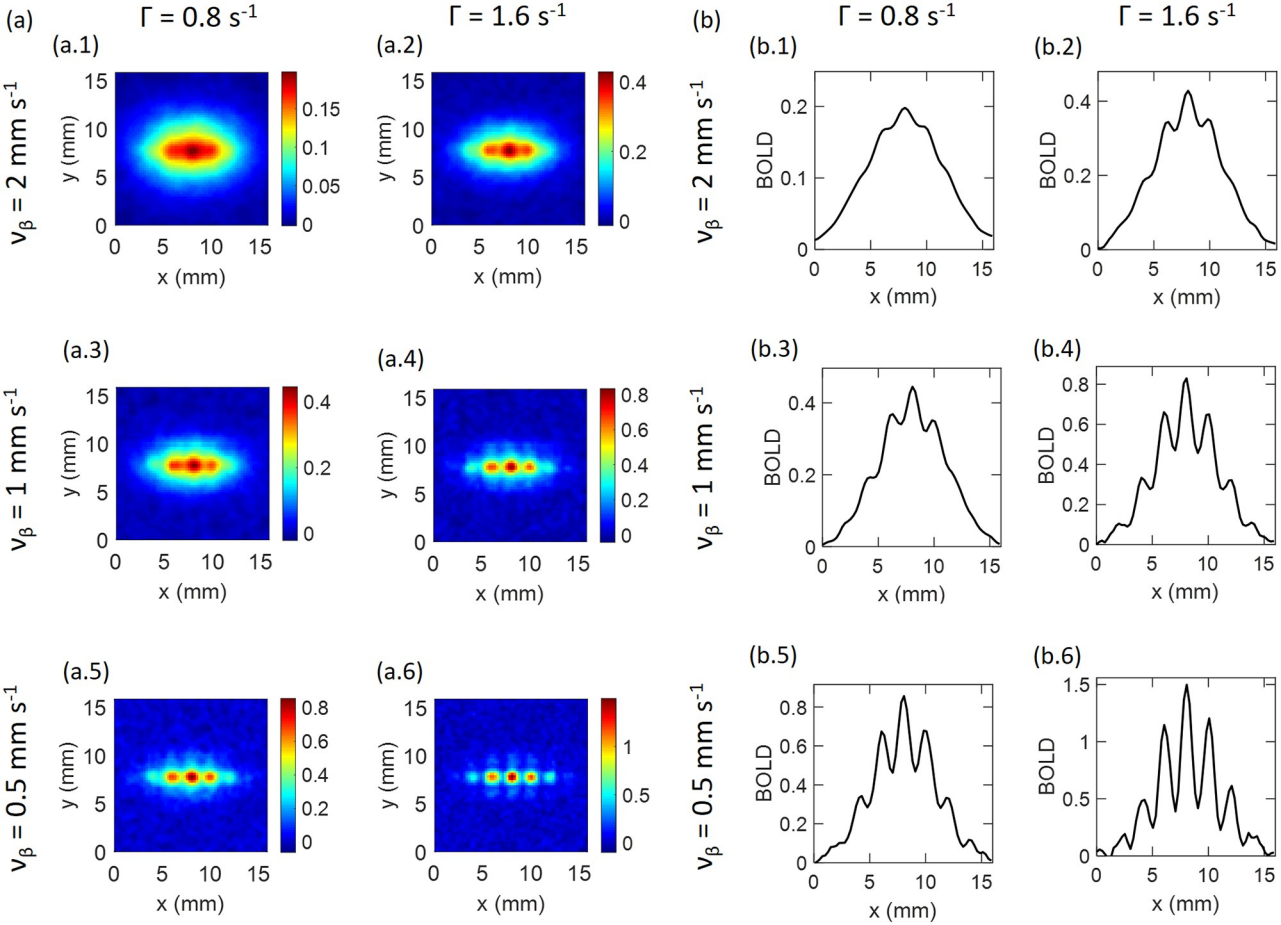
Sensitivity analysis of BOLD response—Noisy BOLD-Wiener. (a) BOLD images for various Γ and *v*_*β*_, as labeled, with OP of 0° and resolution of 0.25 mm in all cases. (b) Profiles of the images in (a) in the *x*-direction.

Two main parameters have been found to influence the form of the hemodynamics and resulting BOLD response: the wave damping rate (Γ) and the propagation speed (*ν*_*β*_). The values estimated in previous work [34] were Γ = 0.8 s^−1^ and *ν_β_* = 2 mm s^−1^, which are our default values. However, these parameters are likely to vary between subjects and brain areas, so we explore their effects. According to Aquino et al. [25], the estimated values of Γ and *ν*_*β*_ vary from 0.2 to 2 s^−1^ and 1 to 20 mm s^−1^, respectively. By considering a wide range of values, we find that lower Γ and higher *ν*_*β*_ than the default values do not show any modulation [i.e., the profile of BOLD and modulation curves are smoother than the results shown in Fig 8(a.1) and 8(b.1)]. Hence, we focus on higher Γ and lower *ν*_*β*_. Fig 8 shows these results for noisy BOLD-Wiener. It is seen that for the default parameters at a resolution of 0.25 mm, the BOLD response in Fig 8(a.1) shows no modulation and the BOLD magnitude is ∼0.2, as seen in Fig 8(b.1), making it difficult to resolve OD and OP features, but *ϵ* ≈ 46%. When the damping is twice as high (Γ = 1.6 mm s^−1^), the modulation increases to approximately 8%, the BOLD magnitude is approximately doubled, OD and OP features are more visible, as seen in Fig 8(a.2) and 8(b.2), and *ϵ* ≈ 36%. Similarly, as *ν*_*β*_ decreases to 1 mm s^−1^, the modulation and BOLD magnitude increase, as shown in Fig 8(a.3) and 8(b.3).

The results are similar for Γ = 1.6 s^−1^ and *ν_β_* = 1 mm s^−1^, and Γ = 0.8 s^−1^ and *ν_β_* = 0.5 mm s^−1^. The modulation increases further to *ξ* ≈ 24%, *ϵ* ≈ 52% and *ξ* ≈ 24%, *ϵ* ≈ 71% as shown in Fig 8(b.4) and 8(b.5), respectively. The BOLD magnitude also increases to approximately 0.8 and OD and OP features become more visible, as seen in Fig 8(a.4) and 8(a.5), respectively. A conjunction of large Γ and low *ν*_*β*_ further increases the modulation and the BOLD magnitude: at Γ = 1.6 s^−1^ and *ν_β_* = 0.5 mm s^−1^, the modulation reaches *ξ* ≈ 52%, *ϵ* ≈ 52% and the BOLD magnitude is close to 1.5, as seen in Fig 8(a.6) and 8(b.6).

**Fig 9:**
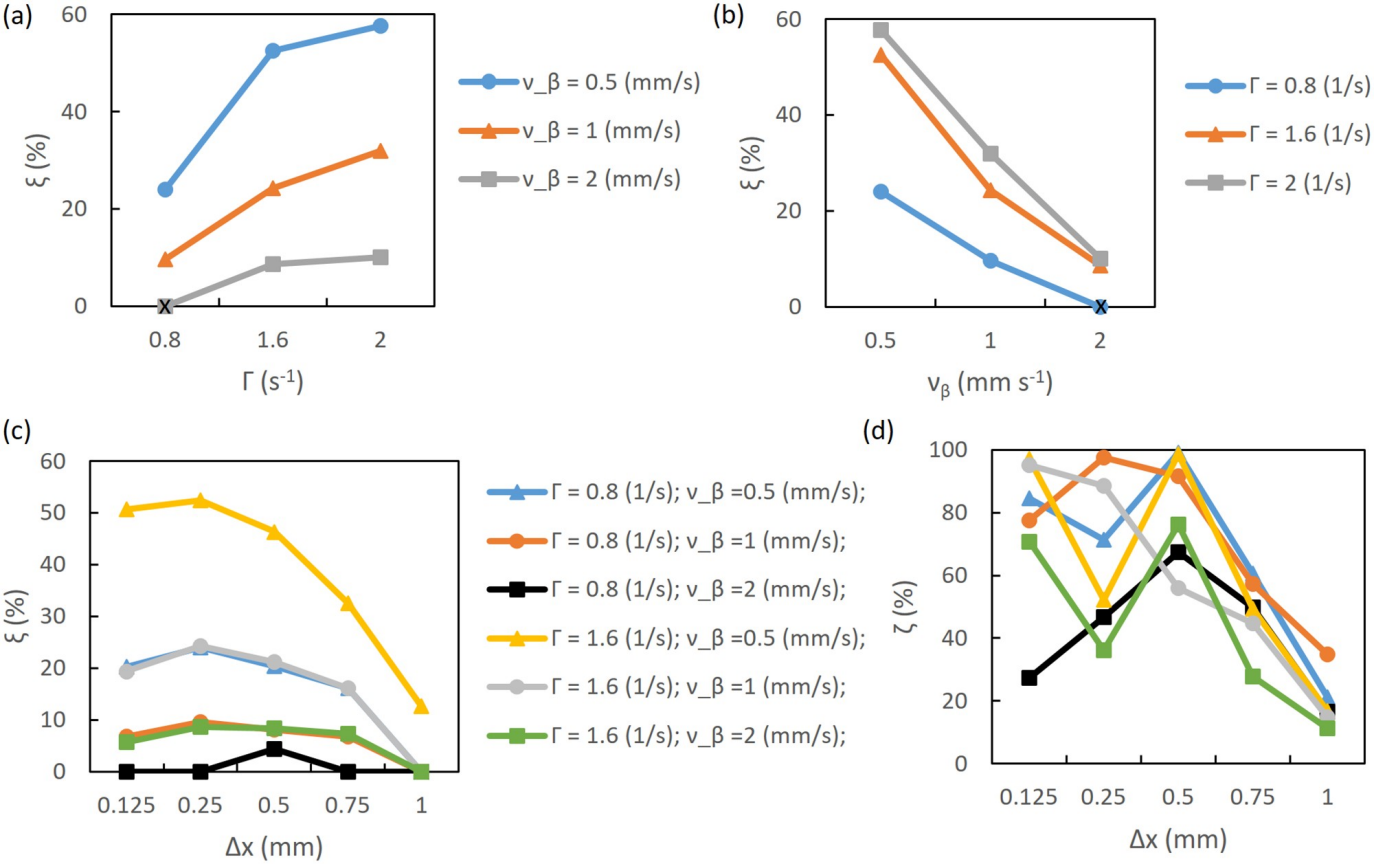
Modulation of noisy BOLD-Wiener from results in Fig 8(b) and modulation of corresponding deconvolved neural activity; OP of 0°. (a) Modulation as a function of Γ, varying *ν*_*β*_ (as shown in the legend), at a resolution of 0.25 mm. (b) Modulation as a function of *ν*_*β*_, varying Γ (as shown in the legend), at a resolution of 0.25 mm. (c) and (d) Modulation as a function of resolution for various Γ and *ν*_*β*_, as shown in the legend; (c) noisy BOLD-Wiener and (d) corresponding deconvolved neural activity. The black symbols represent results for the default case.

Fig 9 summarizes the modulation of the noisy BOLD-Wiener signal for different Γ and *ν*_*β*_. Fig 9(a) and 9(b) explicitly show the behavior that a low *ν*_*β*_ and a large Γ increases the modulation in BOLD response. These figures only show the behavior of noisy BOLD-Wiener; however, the percentage modulation for other types of BOLD response (BOLD, BOLD-Wiener, and noisy BOLD-Wiener) have similar values, because the level of noise is relatively low.

Fig 9(c) shows the effect of measurement resolution Δ*x* on the modulation of the noisy BOLD-Wiener signal for various Γ and *ν*_*β*_, and Fig 9(d) shows the corresponding deconvolved neural activity. For the default case (Γ = 0.8 s^−1^ and *ν_β_* = 2 mm s^−1^), noisy BOLD-Wiener modulation only occurs at a resolution of 0.5 mm, *ξ* ≈ 4%; whereas neural activity modulation occurs for all resolutions and the values vary from *ϵ* ≈ 16% to *ϵ* ≈ 67%. For the cases Γ = 0.8 s^−1^ and *ν_β_* = 1 mm s^−1^ and Γ = 1.6 s^−1^ and *ν_β_* = 2 mm s^−1^, modulation varies from *ξ* ≈ 5% to *ξ* ≈ 9%, and there is no modulation at a resolution of 1 mm; while *ϵ* varies from ≈ 34% to ≈ 97% and ≈ 11% to ≈ 70%, respectively. For the cases Γ = 0.8 s^−1^ and *ν_β_* = 0.5 mm s^−1^ and Γ = 1.6 s^−1^ and *ν_β_* = 1 mm s^−1^, modulation varies from *ξ* ≈ 16% to *ξ* ≈ 24%, for resolutions from 0.125 mm to 0.75 mm; and *ϵ* varies from ≈ 15% to ≈ 99%, for all resolutions. Stronger modulations occur for high damping and low speed, as shown for the case of Γ = 1.6 s^−1^ and *ν_β_* = 0.5 mm s^−1^, where noisy BOLD-Wiener modulation is evident even at 1 mm resolution.

**Fig 10:**
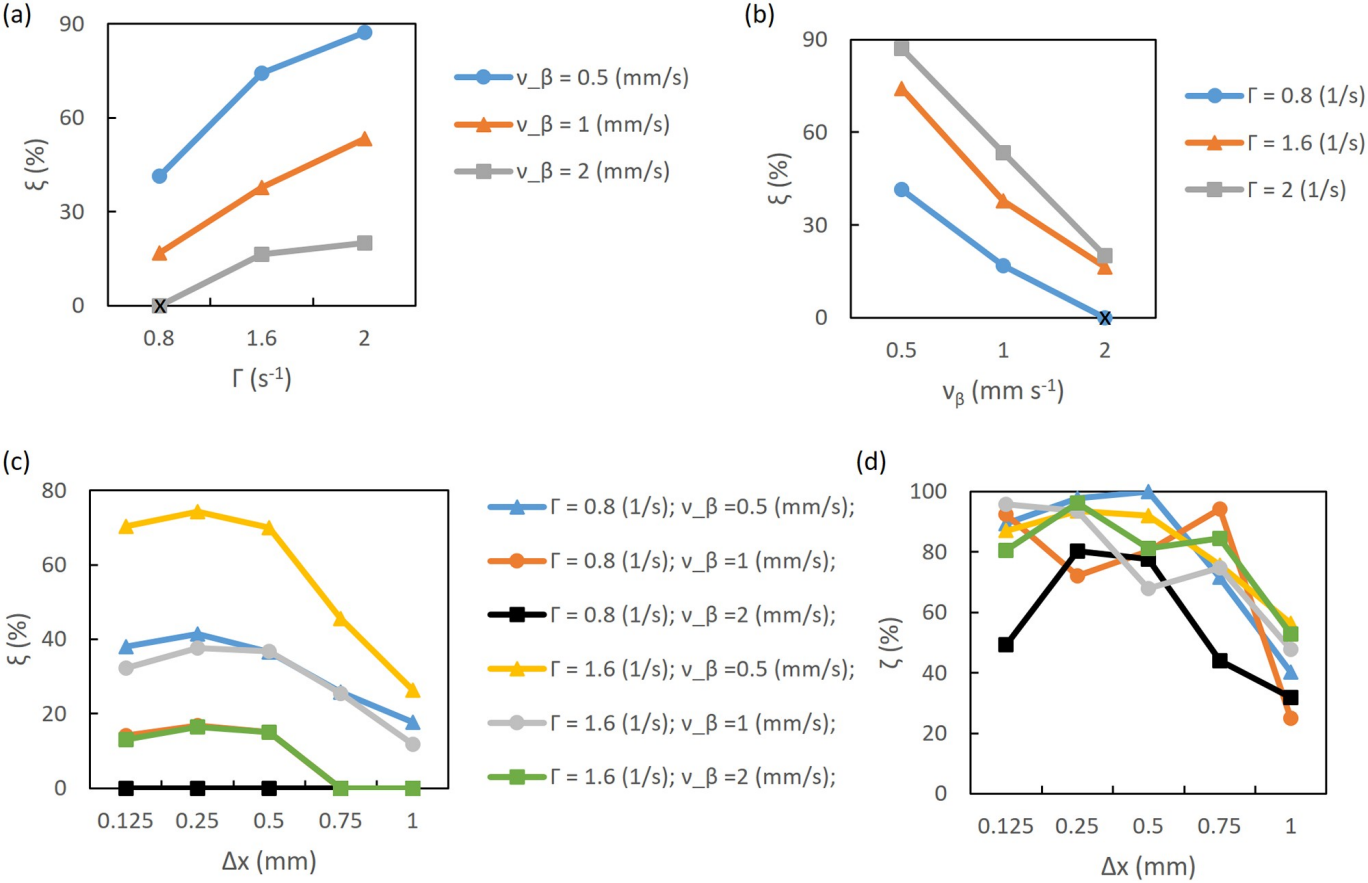
Modulation of noisy BOLD-Wiener at OP of 45°, and modulation of corresponding deconvolved neural activity. (a) Modulation as a function of Γ, varying *ν*_*β*_ (as shown in the legend), at a resolution of 0.25 mm. (b) Modulation as a functions of *ν*_*β*_, varying Γ (as shown in the legend), at a resolution of 0.25 mm. (c) and (d) Modulation as a function of resolution for various Γ and *ν*_*β*_, as shown in the legend; (c) noisy BOLD-Wiener and (d) corresponding deconvolved neural activity. The black symbols represent results for the default case.

Results for OP of 45° are shown in Fig 10. It is seen that the modulation is higher than in the 0° case. As expected, modulation rises when Γ increases [see Fig 10(a)] and *ν*_*β*_ decreases [see Fig 10(b)]. However, for these cases, noisy BOLD-Wiener modulation reaches nearly 87% when Γ = 2 s^−1^ and *ν_β_* = 0.5 mm s^−1^. Comparing Figs 10(c) and 9(c), it can be seen that the curves follow equivalent patterns; however, an increase of *ξ* from 38% to 75% occurs for OP of 45° for the noisy BOLD-Wiener modulation. Although no modulation appears for the default case at 0.5 mm resolution, and to when Γ = 0.8 s^−1^ and *ν_β_* = 1 mm s^−1^ and Γ = 1.6 s^−1^ and *ν_β_* = 2 mm s^−1^ at 0.75 mm resolution, modulation is seen at Δ*x* ≈ 1 mm for *ν_β_* = 0.5 mm s^−1^. Fig 10(d) shows the corresponding deconvolved neural activity in which all resolutions have a positive modulation, with *ϵ* varying from ≈ 25% to ≈ 99%. Therefore, from Figs 9(d) and 10(d), it can be seen that neural activity modulation is positive even for cases where no modulation is evident for the noisy BOLD-Wiener.

## Discussion

Here, a hemodynamic model was used to explore the feasibility of detecting OD and OP features in V1 via fMRI. First, a spatially patchy neural activity was developed to stimulate different OD and OP cells. Then, the model was used to estimate the BOLD response to the above neural activity. Conversely, the model was also used to perform a Wiener deconvolution to recover the underlying neural activity from a measured BOLD response, with and without noise. The main findings of the study are:

(i) OD and OP features can be detected directly from the BOLD response for resolutions of Δ*x* ≈ 0.5 mm. Under optimal conditions, detection is possible for resolutions up to 1 mm, whereas coarser resolutions make detection impossible.
(ii) Wiener deconvolution can be applied to partly denoise the BOLD response and to infer the underlying neural activity. For many cases where OD-OP features cannot be resolved directly from fMRI because of hemodynamic spreading and noise, deconvolution becomes useful, yielding usable resolutions from Δ*x* ≈ 0.125 − 1.0 mm. This includes a broader range of feasible resolutions than the ≈ 0.8 mm optimal resolution found by [20] without Wiener deconvolution.
(iii) The BOLD responses of cells with OPs that have patchy projections at angles near 45° and 135° relative to local OD boundaries have higher modulations than cells with OPs that project at angles near 0° and 90° relative to the OD boundaries, because the distance among the former are higher by a factor of $\sqrt{2}$. Detection of these OPs would constrain the OD-OP map even if other OPs were not resolved.
(iv) The detectability of OD and OP features from the BOLD response also depends on the biophysical properties of cortical tissue. Hemodynamic wave damping (Γ) and speed (*ν*_*β*_) are most relevant in detecting OD-OP features, with low *ν*_*β*_ and high Γ resulting in better detection because they correspond to less hemodynamic spreading.

Overall, our results suggest that current 7T fMRI with resolutions between 0.5 − 0.75 mm, especially combined with deconvolution, should be able to resolve OD-OP features; and that OPs that project at 45° and 135° to OD boundaries are easier to be detected. The use of model-based deconvolution to remove the effects of hemodynamic spreading, combined with Wiener (or similar) filtering to reduce the effects of noise, will be essential to reach resolutions below about 0.5 mm, and to imaging the neural activity and intermediate processes that underlie BOLD. In future work, we will apply our methods and results to actual fMRI experiments, to verify and benefit from the modelling conducted here.
